# Recovery of Olfactory Function After Mepolizumab Treatment in Patients with Chronic Rhinosinusitis with Nasal Polyps: Influence of the Number of Surgeries

**DOI:** 10.3390/biomedicines14071497

**Published:** 2026-07-02

**Authors:** Alda Cardesín, Ana Sogo, Aina Sansa, Mariana Campos, Carlota Rovira, Christian Domingo

**Affiliations:** 1 Rhinology and Sleep Unit, Department of Otorhinolaryngology, Parc Taulí Hospital Universitari, Institut d’Investigació i Innovació Parc Taulí (I3PT-CERCA), Universitat Autònoma de Barcelona (UAB), 08208 Sabadell, Spain; aldacardesin@yahoo.com (A.C.); asansa@tauli.cat (A.S.); marianacamposm@gmail.com (M.C.); crovira.orl@gmail.com (C.R.); 2 Department of Pulmonary Medicine, Parc Taulí Hospital Universitari, Institut d’Investigació i Innovació Parc Taulí (I3PT-CERCA), Universitat Autònoma de Barcelona (UAB), 08208 Sabadell, Spain; sogosagardia@gmail.com

**Keywords:** chronic rhinosinusitis, nasal polyps, mepolizumab, biologic therapies, olfactory disorders, endoscopic sinus surgery

## Abstract

**Background**: Chronic rhinosinusitis with nasal polyps (CRSwNP) is a predominantly type 2 inflammatory disease associated with significant olfactory dysfunction. The real-life effect of mepolizumab on smell recovery and the influence of prior surgery and peripheral eosinophilia remain unclear. **Objective**: Our objective was to evaluate olfactory outcomes after 12 months of mepolizumab in CRSwNP and analyze the impact of previous surgeries and baseline blood eosinophilia. **Methods**: This was a prospective observational study including 33 consecutive CRSwNP patients treated with mepolizumab. Olfactory function was assessed using subjective measures (VAS: Visual Analogue Scale; SNOT-22: Sino-nasal outcome test, item 21) and psychophysical testing (BOT-8: Barcelona Olfactory Test, detection and identification). Nasal Polyp Score (NPS), peripheral eosinophilia, and quality of life were recorded. Patients were stratified by number of prior sinonasal surgeries. **Results**: Significant improvement occurred in all subjective and objective olfactory measures at 12 months (BOT-8 detection: 100% vs. 0%; identification: 71% vs. 0%; VAS: 3 vs. 10; SNOT-22 item 21: 1 vs. 5; all *p* < 0.05). A lower improvement occurred in patients with ≥3 prior; however, this subgroup was small (*p* < 0.001). Baseline blood eosinophilia was not associated with olfactory improvement. Larger baseline polyp size correlated inversely with subjective olfactory gain (r = −0.48; *p* = 0.03). **Conclusions**: Twelve months of mepolizumab improved olfactory function in CRSwNP, especially in patients with fewer prior surgeries. Olfactory dysfunction responds to multifactorial mechanisms beyond blood eosinophilia, including tissue remodeling, nostril obstruction and possible neuroinflammatory mechanisms involving central olfactory pathways, supporting psychophysical test assessment and reinforcing olfaction as a marker of therapeutic response.

## 1. Introduction

Chronic rhinosinusitis with nasal polyps (CRSwNP) is an inflammatory disease of the sinonasal mucosa affecting 2–4% of the general population. It significantly impairs patients’ quality of life and generates a high consumption of healthcare resources [1,2]. Olfactory dysfunction, either hyposmia or anosmia, is a clinical biomarker of this condition and is considered a disabling symptom, with repercussions on nutrition, environmental perception, personal safety, and emotional well-being [3,4].

Olfactory loss in CRSwNP is a multifactorial phenomenon resulting from both mechanical obstruction of the olfactory cleft by polyps and edema, and peripheral type 2 inflammation mediated by eosinophils and cytokines such as IL-4, IL-5, and IL-13, which promote epithelial remodeling and alter olfactory neurogenesis [5,6]. This dual pathway explains why some patients may recover normosmia after adequate control of inflammation and obstruction, whereas in other cases persistent structural damage or irreversible remodeling of the olfactory epithelium may limit recovery even after surgery or biologic therapy [7]. Moreover, central neuroinflammation, a frequently overlooked concept, has been proposed. Recent studies in animal models and systematic reviews in humans suggest that olfactory dysfunction may reflect not only peripheral inflammation but also a sustained central neuroinflammatory component characterized by glial activation and synaptic alterations in the olfactory bulb [8,9].

Improved understanding of pathophysiology has led to the use of biologic therapies targeting type 2 inflammation. Mepolizumab, an anti–IL-5 monoclonal antibody, has demonstrated efficacy in reducing polyp size, improving nasal congestion, and promoting olfactory recovery in clinical trials and real-life studies [10,11]. Other biologics such as omalizumab, benralizumab, tezepelumab, and dupilumab have also shown consistent results in this setting, consolidating the role of olfaction as a key marker of therapeutic response [12,13,14,15,16].

In clinical practice, olfactory assessment combines subjective scales, such as the Sino-nasal outcome test (SNOT-22) item 21 or the Visual Analogue Scale (VAS), with validated psychophysical tests such as the UPSIT, Sniffin’ Sticks, and adapted tools like the Barcelona Smell Test-24 (BAST-24) and the Barcelona Olfactory Test-8 (BOT-8), validated in the Spanish population [17,18]. Although endoscopic sinus surgery has been the mainstay of treatment for decades, several studies have shown that multiple surgical interventions are associated with lower rates of olfactory recovery, supporting a more integrated approach that considers both inflammatory burden and optimal timing for biologic therapy [7,19].

The aim of the present study was to evaluate changes in olfactory function in patients with CRSwNP treated with mepolizumab and to analyze the influence of the number of previous surgeries and peripheral eosinophilia on the magnitude of recovery.

## 2. Materials and Methods

### 2.1. Study Design

This is a prospective, longitudinal observational study that compiles information from the medical records of patients treated in the past and prospectively followed according to the medical care protocol of our unit, conducted in a Unified Airway Diagnostic and Therapeutic Unit (UDTVAU—Spanish acronym) of a university hospital. The primary objective was to evaluate changes in olfactory function in patients with CRSwNP treated with mepolizumab for a minimum of 12 months. Secondary objectives included assessing the effect of the number of previous surgeries, inflammatory burden, and the type of olfactory assessment tool used.

### 2.2. Study Population

All thirty-three consecutive patients with a diagnosis of CRSwNP who initiated biologic therapy for the first time were included. Patients initiated mepolizumab treatment in 2024. Biologic therapy was indicated due to nasal polyposis and/or asthma.

The inclusion criteria were the following:

1 Age ≥ 18 years.
2 Eligibility for biologic therapy for asthma or CRSwNP refractory to conventional medical treatment according to international guidelines [20,21].
3 Fulfillment of Spanish reimbursement criteria.

The exclusion criteria were the following:

1 Post-COVID-19 anosmia.
2 Neurological or psychiatric diseases associated with olfactory dysfunction.
3 Congenital olfactory disorders.
4 Unilateral nasal polyposis of other aetiology.
5 Traumatic brain injury.
6 Follow-up <12 months.

### 2.3. Outcome Measures

The primary efficacy variable was olfactory function measured using the VAS. Other variables included question 21 of the SNOT22 questionnaire regarding smell and the detection and identification of BOT8. The secondary variables studied were quality of life, polyp size, and number of eosinophils in peripheral blood. The primary safety variable was the collection of adverse events attributable to mepolizumab.

### 2.4. Assessments

Assessments were performed at baseline and after 6 and 12 months of treatment. Olfactory function was evaluated using combined subjective and psychophysical tools. Subjective assessment included olfactory VAS (0–10; 0 = normal smell; 10 = complete loss of smell) and SNOT-22 item 21. Psychophysical assessment was performed using the Barcelona Olfactory Test-8 (BOT-8, Barcelona, Spain), which includes detection (DT) and identification (ID) domains.

Quality of life was assessed using the validated Spanish version of the SNOT-22 questionnaire [22]. Nasal polyp size was evaluated through bilateral nasal endoscopy and expressed using the Nasal Polyp Score (NPS, range 1–8). Peripheral blood eosinophil counts were recorded.

Patients were stratified according to the number of previous endoscopic sinonasal surgeries (ESSs) (1, 2, or ≥3).

Criteria for improvement were based on Han [23] and Bachert’s [24] papers: NPS > 1; VAS > 3; SNOT-22 > 8.9. Severity classification followed EPOS criteria: mild 0–3; moderate > 3–7; severe > 7–10 [25].

The olfactometry variables were evaluated according to the following studies: (a) For the BOT8, data from the olfactometry variables (detection and identification) were used, and the criteria of Rojas-Lechuga [18] were applied, which define anosmia as ≤3, (b) hyposmia as 4 to <6, and >6 as normal. (c) The percentage expression (consistent with the data from Cardesín [17]) classifies hyposmia as severe < 40, moderate 40–60%, mild 61–74%, or normal > 75%. (d) Eosinophilia will be assessed according to the results of the REDES study [26].

### 2.5. Sample Size Calculation

An improvement in the VAS >3 was considered clinically relevant. In the SYNAPSE study, around 60% of the patients showed a VAS improvement >3 at month 12 [27].

Thus, we accepted that, in real life, a 40% clinical response was reasonable. Thus, accepting an alpha risk of 0.025 and a power of 0.9 in a two-tailed test, 33 patients are necessary to recognize a statistically significant difference with an initial proportion of 0 and a final proportion of 0.4. A drop-out rate of 0 was anticipated.

### 2.6. Statistical Analysis

The distribution of continuous variables was assessed using the Shapiro–Wilk test. As none followed a normal distribution, all were expressed as median and interquartile range (IQR). The Friedman test was used to assess the overall longitudinal trend across the three time points, and pairwise comparisons against baseline were performed using the Wilcoxon signed-rank test.

The associations among quantitative clinical variables (baseline eosinophil count, polyp size, and recovery of the sense of smell) were analyzed using Spearman’s correlation coefficient. A *p*-value < 0.05 was considered statistically significant.

Statistical analysis was performed using the R software (v. 4.3.1) and the RStudio environment (v. 2023.06.0, Posit Software, Boston, MA, USA).

## 3. Results

### 3.1. Population Characteristics

Thirty-three patients with CRSwNP treated with mepolizumab were included. The median age was 59 years (IQR 50–65); 66.0% were women (n = 22) and 34.0% were men (n = 11). The demographic characteristics are shown in Table 1.

### 3.2. Improvement in Quality of Life and Olfactory Function

**Figure 1:**
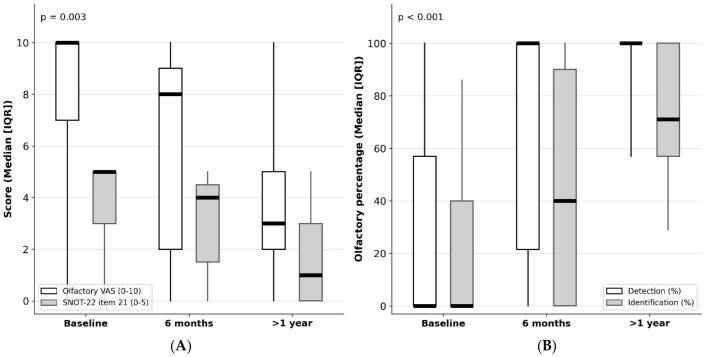
These figures show the temporal progression of SNOT-22 item 21 and VAS (**A**) and odor detection and identification measured using BOT-8 (**B**). The worst VAS value is 10; the best is 0. A decrease in the SNOT-22 value illustrates an improvement. SNOT-22 value ranges between 0 and 110, the higher value being the worst quality of life.

After initiation of mepolizumab, significant clinical improvements were observed across all subjective, psychophysical, and endoscopic measures of disease severity, as well as in quality of life (Table 2 and Table 3, Figure 1). The overall longitudinal trend (Friedman test) was statistically significant for olfactory VAS, SNOT-22 item 21, SNOT-22 total score, and both BOT-8 domains (detection and identification), whereas the trend for absolute eosinophil count did not reach statistical significance (*p* = 0.092), despite a marked reduction at 6 months and 1 year. Pairwise comparisons against baseline (Wilcoxon signed-rank test) showed statistically significant differences at 1 year for these variables (*p* < 0.05), with no significant differences detected at 6 months. The Nasal Polyp Score (NPS) showed a marked and statistically significant reduction compared with baseline both at 6 months and at 1 year.

The primary variables corresponding to the evaluation of the olfactory function are shown in Table 2. The secondary variables are shown in Table 3 and Figure 1.

### 3.3. Evolution of Olfactory Function After Treatment with Mepolizumab (BOT-8 Detection and Identification at 6 and 12 Months)

Psychophysical olfactory testing (BOT-8) showed a progressive improvement in both detection and identification domains. Median (IQR) detection scores increased from 0 (0–57) at baseline to 100 (21.5–100) at 6 months and 100 (100–100) at 1 year; identification scores increased from 0 (0–40) to 40 (0–90) and 71 (57–100), respectively. Both changes were statistically significant overall (Friedman *p* < 0.001) and compared with baseline at 1 year (Wilcoxon *p* < 0.001), with no significant difference detected at 6 months.

### 3.4. Influence of the Number of Previous Surgeries

**Figure 2:**
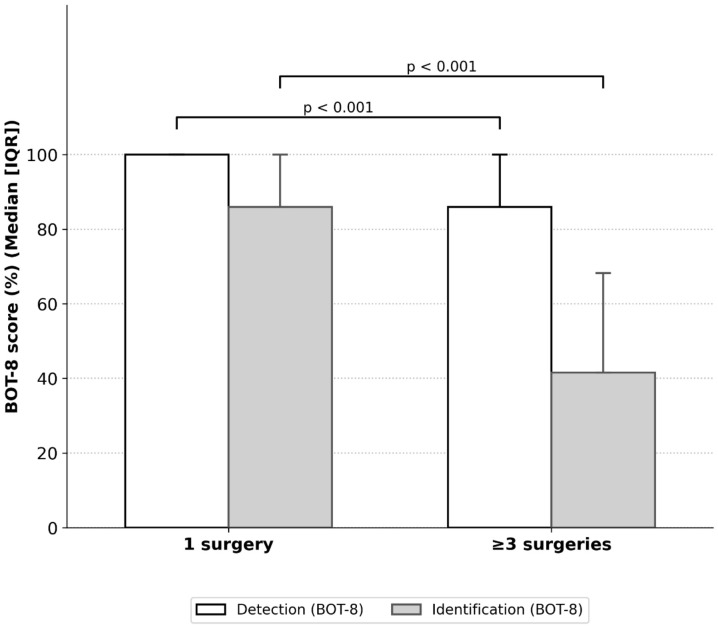
Influence of the number of surgeries on olfactory recovery.

After categorizing the patients according to the number of surgical procedures, the nonparametric Kruskal–Wallis test was used, followed by pairwise comparisons using the Mann–Whitney U test to identify specific differences between subgroups, applying the Bonferroni correction. Subgroup analysis revealed significantly lower psychophysical recovery in patients with ≥3 previous surgeries compared to those with one surgery (detection: 86 [0–100] vs. 100 [100–100]; identification: 41.5 [0–68.25] vs. 86 [71–100]; *p* < 0.001) (Figure 2). No significant differences were observed in subjective scales (SNOT-22 and olfactory VAS).

### 3.5. Relationship with Eosinophils

This association was examined using Spearman’s correlation coefficient (rs). No statistically significant correlation was observed between baseline peripheral blood eosinophil levels and olfactory recovery after mepolizumab treatment for BOT-8 identification (rs = −0.27, *p* = 0.483) and for BOT-8 detection (rs = 0.30, *p* = 0.064).

### 3.6. Relationship with Polyp Size

A significant inverse relationship was identified between baseline nasal polyp size and improvement in SNOT-22 item 21 (r = −0.48; *p* = 0.03).

### 3.7. Treatment Safety

No drug-related side effects were detected.

## 4. Discussion

Olfactory function is an essential component of daily life, linked not only to sensory perception but also to personal safety, enjoyment of food, and emotional and social well-being [4,28]. Its loss has a significant impact on quality of life and has been associated with social isolation and depressive symptoms [4,28]. In recent years, interest in this field has increased, partly due to the COVID-19 pandemic and, more structurally, due to the recognition of its impact in chronic diseases such as chronic rhinosinusitis with nasal polyps (CRSwNP) [29,30].

Traditionally, the assessment of olfactory function in clinical practice has relied on subjective scales such as the Visual Analogue Scale (VAS) or item 21 of the SNOT-22 questionnaire. However, these tools have limitations due to patients’ tendency to under- or overestimate their olfactory abilities [18]. For this reason, it is recommended to complement subjective assessment with objective psychophysical tests. Among internationally validated methods are the UPSIT and Sniffin’ Sticks tests, while, in our setting, specific tools such as the Barcelona Smell Test-24 (BAST-24) have been developed, establishing normative parameters in the Spanish population [17]. More recently, the Barcelona Olfactory Test-8 (BOT-8) has been validated as a brief, sensitive, and patient-accepted tool suitable for routine clinical practice [18]. This justified its use in the present study, which combines subjective and objective olfactory assessment, in contrast to other studies with greater methodological limitations.

From a therapeutic perspective, although endoscopic sinus surgery has been the cornerstone of CRSwNP management for decades, increasing evidence suggests that a higher number of surgical interventions in a patient is associated with poorer olfactory recovery. This finding highlights the need for integrated therapeutic strategies that optimize both the timing of surgery and the introduction of biologic therapies [7,11,23].

In CRSwNP, olfactory dysfunction is one of the most persistent and disabling symptoms. Beyond its functional and sensory relevance, olfactory impairment has been associated with a significant impact on the neurocognitive domain. According to Gao et al., 2025 [9], patients with CRSwNP and olfactory dysfunction have a higher risk of cognitive impairment, which may further contribute to the overall deterioration of quality of life.

The pathophysiology of olfactory dysfunction in CRSwNP combines mechanical mechanisms, such as obstruction of the olfactory cleft by polyps and edema, with inflammatory processes mediated by type 2 cytokines, particularly interleukin-5, which are responsible for epithelial remodeling and neuroepithelial damage [31]. This dual mechanism explains the variability in treatment response, whereby some patients recover normosmia after adequate inflammatory control, while others develop irreversible deficits even after multiple medical or surgical treatments [7].

The advent of biologic therapies has modified the therapeutic approach to this disease. Several monoclonal antibodies marketed for asthma have demonstrated efficacy in reducing polyp size, improving nasal congestion, and promoting olfactory recovery in both clinical trials and real-world studies [10,11,12,13,14,16]. In addition, recent real-life studies have confirmed these findings. García-Piñero et al., 2025 [32], reported sustained improvements in symptom burden and quality of life in a multicenter Spanish cohort treated with mepolizumab, including olfactory function, reinforcing its applicability in routine clinical practice. Similarly, Bernstein et al., 2024 [33], highlighted parallel benefits on both upper and lower airways in patients with CRSwNP and comorbid asthma, a relevant finding considering that more than 90% of patients in our cohort presented this association.

The SYNAPSE clinical trial [27] demonstrated sustained improvement in olfactory perception assessed by subjective scales (VAS and SNOT-22) after 52 weeks of mepolizumab treatment. Galletti et al. [34], in a multicenter Italian cohort, used psychophysical tests (Sniffin’ Sticks) and observed a mean gain of seven points in the TDI score after one year of follow-up, reinforcing the validity of objective approaches in olfactory monitoring. In Spain, Domínguez et al. evaluated 52 patients treated with mepolizumab for six months and detected significant improvements in subjective scales such as olfactory VAS and SNOT-22 item 21 [35]. More recently, Domínguez-Sosa et al. reported sustained and progressive improvement in olfactory function at 6, 12, and 24 months in a real-world cohort of CRSwNP patients treated with mepolizumab, supporting the long-term durability of olfactory recovery observed in both clinical trials and real-life studies [36].

In our cohort, treatment with mepolizumab for at least 12 months was associated with a significant improvement in olfactory function, assessed by both subjective measures and psychophysical tests, in line with previously published data and reinforcing its role in routine clinical practice. While subjective scales (VAS and SNOT-22) showed moderate improvements, BOT-8 demonstrated more pronounced gains in both the detection and identification of odors. This finding is consistent with studies indicating that self-assessment of olfactory function tends to underestimate the true magnitude of the deficit Sorokowska et al. [28]. Recovery of odor identification, which involves higher cognitive processing, suggests a deeper functional effect of treatment.

The choice of BOT-8, recently validated in the Spanish population [18], represents an added value due to its brevity and acceptability, complementing previous experience with BAST-24 [17], which is longer and less practical for routine follow-up of patients receiving biologic therapy, although in other aetiologies the information obtained with this last test is more widespread.

Our findings indicate that greater baseline polyp burden, assessed using the Nasal Polyp Score, is significantly associated with poorer olfactory recovery indirectly represented by SNOT-22 item 21 after treatment with mepolizumab. This statistically significant relationship is consistent with previous studies, suggesting that persistent mechanical obstruction of the olfactory cleft, together with more intense chronic inflammation, may limit functional reversibility even after effective modulation of type 2 inflammation. Although direct evidence specifically evaluating the relationship between nasal polyp size reduction and improvement in psychophysical olfactory tests during mepolizumab treatment remains limited, previous studies using objective olfactory assessment tools have suggested a similar association. In the SYNAPSE-related olfactory analysis, Mullol et al. [37] demonstrated significant improvement in smell outcomes assessed with UPSIT, together with reductions in disease severity and polyp burden, while real-world studies with mepolizumab using psychophysical tools such as Sniffin’ Sticks have also reported parallel improvements in olfactory function and Nasal Polyp Scores [34]. Our findings are consistent with the existing evidence suggesting that reduction in inflammatory and polyp burden may contribute to olfactory recovery. The EPOS 2023 guidelines and the POLINA 2.0 consensus also recognize polyp size as an indirect marker of inflammatory burden and as a prognostic factor in response to biologic therapy [19,20]. Therefore, baseline polyp burden should be considered a key element in the selection and monitoring of patients who are candidates for biologic treatment, particularly when olfactory recovery is a priority therapeutic goal.

Our results also suggest that the number of previous surgeries may influence olfactory recovery. Patients with three or more surgical interventions exhibited less improvement in psychophysical tests compared with those who had undergone one or two surgeries. This difference was not detected using subjective scales, suggesting a possible cumulative effect of structural or inflammatory damage. However, this observation should be interpreted with caution, as only two patients in our cohort belonged to the subgroup with ≥3 previous surgeries, which limits the statistical robustness and generalizability of this comparison. These findings are consistent with recent series [7,38]. In this regard, studies such as that by Homøe et al. 2025 [39] have shown that combining mepolizumab with surgery provides superior benefits compared with biologic therapy alone, particularly in terms of polyp control and olfactory recovery. Additionally, Garvey et al. [11] emphasized the importance of optimizing the timing of biologic therapy in relation to surgery to maximize clinical efficacy, supporting a sequential and individualized therapeutic approach in refractory disease.

Joint analysis of these variables showed that patients with a higher number of surgical interventions did not necessarily present higher baseline eosinophilia, supporting the hypothesis that factors such as chronic tissue remodeling, disease duration, and prior treatments—including systemic corticosteroids and surgery—may modulate the systemic inflammatory profile beyond the number of procedures performed. This finding is consistent with studies that have not identified a direct correlation between eosinophilia and the number of previous surgeries, suggesting that inflammatory burden may fluctuate depending on disease stage and therapeutic history [40]. Conversely, other studies have proposed that persistent or recurrent eosinophilia is associated with a higher risk of recurrence and, consequently, a greater number of surgical interventions [10,41,42].

In the present study, the recurrence of peripheral eosinophilia was not evaluated. However, no significant association was found between baseline eosinophil levels and the magnitude of olfactory recovery measured by either VAS or BOT-8. This suggests the concept that olfactory dysfunction in CRSwNP is influenced by multiple mechanisms beyond the initial peripheral inflammatory profile, including structural alterations, irreversible neuroepithelial damage, and individual susceptibility factors.

Experimental murine models and human biopsy studies of the olfactory epithelium have shown that infiltration by eosinophils and mast cells, together with the overexpression of cytokines such as IL-4, IL-5, IL-13, and eotaxin, induces profound alterations in the olfactory mucosa [6,43]. These changes include a reduction in immature olfactory neurons (GAP43+), disorganization of the neurosensory epithelium, and decreased expression of genes associated with neurogenesis such as NEUROG1, TRP63, and RGS1. Such alterations may compromise the regenerative capacity of the olfactory neuroepithelium, even when the inflammatory stimulus is adequately controlled. This impact of type 2 inflammation on olfactory neuroepithelial structure and function has also been confirmed in animal models, such as those developed by Zhang and colleagues, who demonstrated that chronic eosinophilic inflammation induces sustained microglial activation and neurotransmission alterations in the olfactory bulb, contributing to the olfactory dysfunction observed in CRSwNP. These experimental findings may help explain why some patients with high inflammatory burden or chronic tissue damage exhibit partial or absent olfactory recovery despite an adequate overall clinical response to biologic therapy [8].

Although biologic treatments have demonstrated efficacy in improving olfactory dysfunction in CRSwNP, not all patients achieve complete recovery. In those with persistent anosmia and multiple previous surgeries, structural damage and remodeling of the olfactory mucosa may result in irreversible functional loss, regardless of current inflammatory control. Conversely, in patients without prior surgery and/or with preserved baseline olfactory function, a potential component of central neuroinflammatory involvement has been hypothesized, as suggested by Zhang et al., who described glial activation and disruption of the synaptic microenvironment in the olfactory bulb beyond peripheral inflammation [8]. In these cases, preservation of olfactory function may represent a therapeutic opportunity, as olfactory dysfunction has been proposed as an early marker of neurocognitive involvement [9].

The olfactory system is unique among sensory systems, as its afferent pathways project directly from the olfactory bulb to cortical and limbic regions such as the piriform cortex, amygdala, and hippocampus without thalamic relay. This feature makes it a direct channel of cortical stimulation with strong links to memory, emotion, and cognition. Therefore, while improvement in olfactory detection may occur rapidly after nasal decongestion and partial resolution of peripheral inflammation, odor identification—which requires more complex central processing—may recover more slowly and depend largely on subsequent olfactory training that promotes cortical neuroplasticity. In this context, persistent olfactory symptoms may not reflect active peripheral inflammation alone but rather potential central synaptic dysfunction secondary to neuroinflammation [8].

Consequently, olfactory recovery in CRSwNP should be approached in a multifactorial manner, not only by improving airway obstruction and controlling type 2 inflammation, but also by considering the possible contribution of central olfactory pathways, the cumulative impact of disease, and the potential for synaptic regeneration through targeted interventions such as structured olfactory training. Finally, regarding treatment tolerance, no drug-related side effects were detected.

The present study has limitations that restrict the generalizability of the results, including the absence of a contemporary control group and its single-center design. In addition, the relatively small sample size in some subgroup analyses, such as patients with ≥3 previous endoscopic sinus surgeries, limits the statistical robustness of these comparisons. However, the sample size calculation, the prospective nature of the study, follow-up of at least 12 months, and use of validated assessment tools strengthen the robustness of the findings.

## 5. Conclusions

In conclusion, we believe that the combination of subjective and objective variables improves the assessment of the severity of olfactory dysfunction in patients with CRSwNP. The improvement of peripheral inflammation in the nasal mucosa, both anatomically and mechanistically through treatment with mepolizumab, is accompanied by improvements in olfactory function. Patients with a higher burden of previous surgeries appeared to show less improvement in psychophysical olfactory outcomes; however, this observation should be interpreted cautiously due to the small size of this subgroup. A subset of patients appears to be refractory to mepolizumab treatment, potentially due to mechanisms beyond peripheral inflammation alone. Among these, neuroinflammatory processes at the central nervous system level have been hypothesized as a possible contributing factor that could help explain the therapeutic limitations observed in some patients.

## Figures and Tables

**Table 1:** Baseline demographic characteristics of the cohort (n = 33).

Variable	Valor
Age, years (median value [IQR])	59 (50–65)
Female sex, n (%)	22 (66.0)
Male, n (%)	11 (34.0)
Asthma comorbidity, n (%)	31 (93.9)
Only CRSwNP ^1^, n (%)	2 (6)
No prior ESS ^2^, n (%)	6 (18)
1 prior ESS ^2^, n (%)	17 (51)
2 prior ESS ^2^, n (%)	8 (24)
≥3 prior ESS ^2^, n (%)	2 (6)
Baseline eosinophils, cells/μL (median value, [IQR])	685 (402–905)

^1^ CRSwNP: chronic rhinosinusitis with nasal polyps; ^2^ ESS: endoscopic sinonasal surgeries.

**Table 2:** Primary variables.

Assessment	Pre-Treatment	6 Months Post-Treatment	1 Year Post-Treatment	*p*-Value *
BOT8—Detection (%)	0 (0–57)	100 (21.5–100)	100 (100–100) **	<0.001
BOT8—Identification (%)	0 (0–40)	40 (0–90)	71 (57–100) **	<0.001
Olfactory VAS (0–10)	10 (7–10)	8 (2–9)	3 (2–5) **	0.013
SNOT-22 (item 21)	5 (3–5)	4 (1.5–4.5)	1 (0–3) **	0.003

This table includes both subjective variables (olfactory VAS and question 21 of the SNOT-22 questionnaire) and psychophysical measures (detection and identification from the BOT-8 test). Data are expressed as median (interquartile range). * Global *p*-values represent the longitudinal trend across the three time points (Friedman test). ** Statistically significant difference vs. baseline at 1 year only (Wilcoxon signed-rank test, *p* < 0.05).

**Table 3:** Secondary variables.

Assessment	Pre-Treatment	6 Months Post-Treatment	1 Year Post-Treatment	*p*-Value *
NPS (0–8)	5 [2.25–6]	1 (0–2.5) †	1 (0–3) †	0.016
Eosinophils, cells/μL	685 (402.5–905)	40 (25–50)	50 (30–60)	0.092
SNOT-22 total score	34 (25–66)	12 (6.5–18.5)	15 (10–32) **	0.001

This table includes nasal polyp size (Nasal Polyp Score—NPS), calculated as the sum of the scores from each nasal cavity, absolute peripheral eosinophil count, and quality of life measured by the SNOT-22 questionnaire. Data are expressed as median (interquartile range). * Global *p*-values represent the longitudinal trend across the three time points (Friedman test). † For NPS, the reduction vs. baseline was statistically significant both at 6 months (*p* = 0.031) and at 1 year (*p* = 0.016) based on Wilcoxon signed-rank test. ** Statistically significant difference vs. baseline at 1 year only (Wilcoxon signed-rank test, *p* < 0.05).

## Data Availability

The original contributions presented in this study are included in the article. Further inquiries can be directed to the corresponding author.

## Abbreviations

The following abbreviations are used in this manuscript:

BAST-24	Barcelona Smell Test-24
BOT-8	Barcelona Olfactory Test-8
CRSwNP	Chronic rhinosinusitis with nasal polyps
DT	Detection
EPOS	European Position Paper on Rhinosinusitis and Nasal Polyps
ESS	Endoscopic sinonasal surgery
ID	Identification
NPS	Nasal Polyp Score
SNOT-22	Sino-nasal outcome test
UDTVAU	Unified Airway Diagnostic and Therapeutic Unit (spanish acronym: Unidad de Diagnóstico y Tratamiento de Vía Aérea Única)
VAS	Visual Analogue Scale
